# The effect of survival pressure-based defector reward behavior on cooperation in spatial prisoner’s dilemma games

**DOI:** 10.1371/journal.pone.0311612

**Published:** 2024-11-19

**Authors:** Xuechao Zhang, Shichang Lu

**Affiliations:** 1 School of Business Administration, Liaoning Technical University, Huludao, Liaoning, China; 2 University of Science and Technology Liaoning, Anshan, Liaoning, China; Teesside University, UNITED KINGDOM OF GREAT BRITAIN AND NORTHERN IRELAND

## Abstract

Research has shown that rewarding behavior can greatly facilitate the occurrence of cooperation in social dilemmas. Yet rewards entail costs, making the reward itself an altruistic behavior. The reasons for adopting rewarding behaviors then become an interesting matter, so we propose a game model in which defectors are pressured by survival to adopt rewarding behaviors. Research suggests that defector reward strategies can be used as a transition strategy for defectors to alleviate survival stress and promote cooperation in the spatial prisoner’s dilemma. A small survival pressure threshold will make it altogether easier for defectors to adopt a strategy that rewards defection and thus tempts the emergence of cooperators in their neighborhood. In addition, the effect of the payment cost *α* and the reward amount *β* of the rewarding behavior on the evolution of the system will be limited by the temptation b of the defector, and the effect of each parameter on the promotion of cooperation in the system is not linear. That is, when *α* is fixed, b and *β* can still optimize the level of cooperation in a given combination. The same holds for *α* and *β* when b is fixed.

## Introduction

Altruistic behavior has always been present and evolving within in the society we live in. The reasons for its emergence and the process of its evolution have been one of keys to the study of the Numerous disciplines [1–6]. But altruistic behavior will ultimately be based more or less on the loss of self-interest and the achievement of others. And the biological instinct is indeed self-interested, which leads to social dilemmas.

For further research, evolutionary game theory has been proposed and rapidly developed as a separate discipline [7–10]. Various types of dilemmas are also reduced to the most basic game models to be studied. For example, the prisoner’s dilemma game(PDG), the public goods game(PGG), the snowdrift game(SG), etc. [11–20]. PDG requires players to take cooperation (*C*) or defection (*D*) independently before they know their opponent’s strategy. And the combination of player and opponent strategies will directly determine their respective payoff. And defectors have a definite advantage over cooperators. This makes it interesting to see how to facilitate the emergence of cooperative strategies in that game. The related research in this paper is also based on the spatial prisoner’s dilemma.

In order to study social dilemmas and the emergence of cooperation more closely to reality. Nowak and May first put the Prisoner’s Dilemma game into a lattice network in 1992 [21]. They explored the spatial game process under different neighbor matrices by using metacellular automata to simulate the cover process. In their study, spatial structure can facilitate cooperation due to the aggregation behavior of cooperators. Since then research based on spatial reciprocity in evolutionary games has started to become popular [7, 22–24]. Rewards can promote an increase in the number of cooperators in a group by rewarding those players who adopt cooperative strategies [25, 26]. And the penalty mechanism will punish those players who adopt a defection strategy, making them less profitable and thus promoting cooperation [27, 28]. In addition, mechanisms such as such as Reputation [25, 26], aspiration [29, 30], memory effect [31] and teaching ability [14, 25]. They have also been proposed and further studied by related scholars.

Pruitt, Dean G et al. decomposing the Prisoner’s Dilemma game and examining the subtleties between reward structure and cooperation [32]. Babes et al. introduced reward shaping to the Prisoner’s Dilemma game, where individuals shaped by the use of social rewards can lead to encourage their opponents to cooperate [33]. Rau´l Jime´nez et al. propose a shared reward mechanism that allows cooperators to receive additional gains in the second round while defectors cannot [34]. Looking at rewards and punishment alone, there is really no fundamental difference between the two, with penalties being more like negative rewards and vice versa. There is no doubt about their role in facilitating cooperation, but the question is one of who pays for the costs involved [35]. Subsequently Wang et al. explored Tax-based pure punishment and rewards based on the tax system, and they found that punishment and rewards by way of tax payment would be superior to the pure case [36]. Sun et al. have come up with an enhanced version of the defection strategy they call manipulators. They penalize defectors and reward cooperators during the game making a combination of pure reward and punishment [19]. Inspired by the above paper’s definition of pure reward behavior, this paper starts from the motivation and reasons for players to adopt reward behavior. From there, it takes into account the individuals are burdened with survival cost pressures in the gaming process, which will result in them not being able to survive in an environment where there is no gain all the time. This seems to provide an explanation for the motivation of rewarding behavior. Thus, in this paper, we consider the peer competition relationship and combine it with the real-life social phenomenon of sellers competing with each other for consumers, and propose a model in which defectors adopt rewarding behaviors based on the pressure of survival. The defector, due to the fact that he has been within the defector group for a long period of time, then he will be faced with great pressure to survive by adopting non-pro-social behaviors and no gain, and therefore will have to resort to rewarding behaviors in order to attract the emergence of peripheral cooperators. Studies have shown that defectors under survival pressure are forced to adopt rewarding behaviors thereby increasing the level of cooperation in the group. However, this rewarding behavior only works as an intermediate transition strategy, and when defectors are surrounded by more cooperators, they are exposed to the pursuit of higher benefits. The simulation results were used to analyze the effect of variables such as survival pressure threshold *U*_*s*_, reward amount *β*, and reward cost *α* on the evolution of spatial prisoner’s dilemma species cooperation.

The details of this paper are organized as follows. In Section 2, we create a defector Rewards Based on survival pressure model. In Section 3, we analyze and discuss the results of computer simulations of the model and some wonderful discoveries are explored. In the last section, we summarize our research results and findings.

## Model analysis

The model is built on a structural population on a square lattice with periodic boundary conditions. The size of the population is *N* = *L* × *L*(*L* = 100). PDG is used as the basic game model to describe the interaction between players. Players in a game choose a strategy from three options: cooperation (*C*), defection (*D*) and Defection with reward behavior (*RD*). According to existing analyses, defectors with rewarding behaviors will pay a cost *α* to reward cooperators who interact with them. And the cost *α* will remain between 0 − 1 to ensure the game is played more closely to the reality of the situation. The higher the cost *α*, the more the defectors will lose by going for the rewarded behavior. Cooperators are rewarded with *β*. This reward *β* actually represents compensation for individuals for adopting cooperative behavior. It will vary between 0 and 1 and represents the gains that can be made when a cooperator is with a defector with rewarding behavior, so it is critical to the ability of the cooperator to emerge. The simplified game matrix matrix M of the three strategy games is as follows:

$$\begin{array}{c} M=(\begin{array}{ccc} 1 & 0 & \beta \\ b & 0 & 0\\ b-\alpha & 0 & 0 \end{array}) \end{array}$$ (1)

During the game, player i’s strategy can be expressed as

$$\begin{array}{c} S_{i}=(\begin{array}{c} 1\\ 0\\ 0 \end{array}),(\begin{array}{c} 0\\ 1\\ 0 \end{array}),(\begin{array}{c} 0\\ 0\\ 1 \end{array}). \end{array}$$ (2)

The payoff from player i’s interaction with his neighbor player j is *p*_*ij*_

$$\begin{array}{c} p_{ij}={S_{i}}^{T}MS_{j} \end{array}$$ (3)

The payoff *P*_*i*_ that a player *i* accumulates by interacting with his four nearest neighbors, and the total payoff is calculated as

$$\begin{array}{c} P_{i}=\sum_{j\in G_{i}}p_{ij} \end{array}$$ (4)

where *G*_*i*_ is the set of neighbors of *i*.

Based on the matrix *M* in Formula 1, it is clear that the gains of defectors who adopt rewarding behaviors will not be dominant. This means that purely gaming behavior no defector would act this way. However, considering the effect of environmental survival factors, when an individual is completely among defectors (all of a player’s neighbors are defectors), then he will be in a state of no gain for a long time. The pressure of survival forces him to adopt such rewarding behavior to attract the emergence of cooperators around him.

Thus, we introduce a variable *U* to characterize the degree of pressure on players to survive. The player’s survival pressure comes from this hostile social behavior of choosing the defection strategy themselves and their own gains. It will change as the gaming process is repeated, consistent with co-evolutionary rules. Specifically, when player i adopts a defection strategy in round t of the game and his payoff is 0, then his survival pressure will increase by 1. Player i’s survival pressure a at time step t will follow formula 5

$$\begin{array}{c} U_{i}\left(t\right)=\{\begin{array}{ccc} U_{i}\left(t-1\right)+1 & & if\;S_{i}\left(t\right)=(\begin{array}{c} 0\\ 1\\ 0 \end{array})\&\;P_{i}=0\\ 0 & & else \end{array} \end{array}$$ (5)

**Fig 1:**
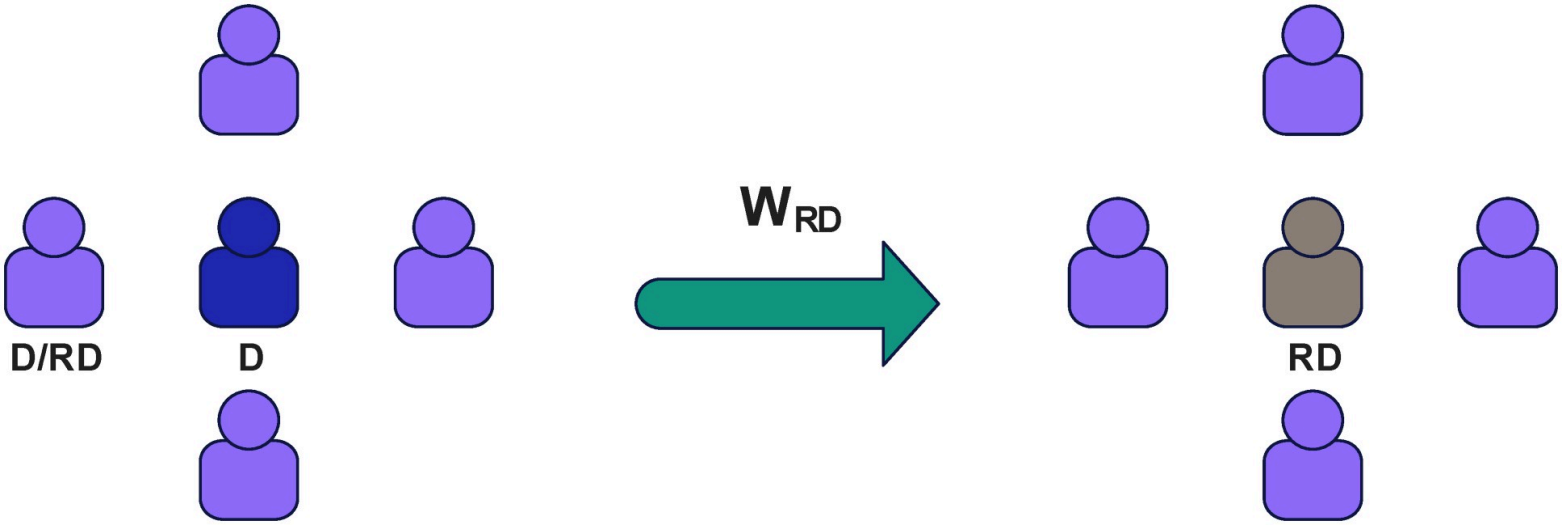
When a defector is in the above environment (surrounded by D or RD) his survival pressure builds up. When the survival pressure of player i is greater than the survival pressure threshold *U*_*s*_, he will directly have a probability *W*_*RD*_ of being directly forced to shift to a defection strategy with rewarding behaviors.

Survival pressure will play into the process of updating the player’s strategy. For this reason we have drawn Fig 1 to visualize the process. When the survival pressure of player i is greater than the survival pressure threshold *U*_*s*_(*U*_*s*_ stands for is a special state of existential stress, which represents the maximum state of stress that an individual can withstand in the current environment. Once the player’s current pressure exceeds this value, it means that the player’s survival is threatened in a long period of time with no profit, and he will start to try to make changes to change his own survival status quo, so as to make his own profit. the larger the *U*_*s*_, it means that the player’s ability to withstand the pressure of survival is stronger, and it is less likely to change his own situation by changing his own strategy.), he will directly have a probability *W*_*RD*_ of being directly forced to shift to a defection strategy with rewarding behaviors, otherwise he will follow the Fermi rule for strategy updating. The exact process will follow formula 6.

$$\begin{array}{c} S_{i}\left(t+1\right)=\{\begin{array}{ccc} \overset{W_{RD}}{\to }(\begin{array}{c} 0\\ 0\\ 1 \end{array}) & & U_{i}\left(t\right)>U_{s}\\ \overset{W_{ij}}{\to }S_{j}\left(t\right) & & U_{i}\left(t\right)\leq U_{s} \end{array} \end{array}$$ (6)

In the above formula, *W*_*RD*_ is the forced survival pressure to switch to a reward defection strategy probability, which will follow the formula 7.

$$\begin{array}{c} W_{RD}=\frac{U_{i}\left(t\right)-U_{s}}{U_{s}} \end{array}$$ (7)

The probability *W*_*ij*_ ∈ [0, 1] that player i randomly chooses a neighbor j to learn and succeeds is

$$\begin{array}{c} W_{ij}=\frac{1}{1+e^{\left[(P_{i}-P_{j})/K\right]}} \end{array}$$ (8)

where *K* represents the amplitude of the noise. It takes the value of 0.1.

Finally, computer simulations were performed in a synchronized update fashion, where the game was iterated forward on a network of squares with L = 100. At each time step, all individuals traverse the above process. To be worth noting. The final displayed cooperation fraction *ρ*_*c*_ is the average taken from the last 5 steps of the 10-step simulation. In addition, to ensure proper accuracy, each final displayed data has more than 30 independent realizations.

## Results

The effect of each of the important variables in the model on the level of cooperation *ρ*_*c*_ is displayed in the heat map in Fig 1. Each column from left to right represents the survival stress threshold *U*_*s*_ = 1, 3, 5. Each row from the top to the bottom controls the invariant quantity separately for *β* = 0.5, *α* = 0.1, *b* = 1.1. The effect of survival stress thresholds on the level of cooperation in the group was significant. The level of cooperation was higher at *U*_*s*_ = 1 than at *U*_*s*_ = 5, controlling for all other parameters being equal, and the level of cooperation at *U*_*s*_ = 3 was in between. This suggests that the survival pressure suffered by players can easily reach this criterion when the survival pressure threshold is small, thus making it easier for defectors to shift to a strategy of defection with rewarding behavior. Thus tempting cooperative players in the group to emerge and maintain at a higher level.

By looking at the first line of the picture we can see that the red areas appear in regions where the temptation to defect is low or the cost of the reward is low. This is similar to the conclusions drawn from previous studies that a defector’s advantage decreases when the temptation to defect is low, whereas a small reward cost increases the frequency of rewarding behaviors, both of which will contribute to the emergence and maintenance of cooperative behaviors. Below we focus on the second row of images, where the defection temptation is not to maintain its original monotonicity, having kept *α* = 0.1. As the defection temptation increases cooperation first decreases and then increases again to a high level and then decreases rapidly. And the value of the bonus *β* has an effect on the appearance of this small peak. The warm-colored bar area in the figure with the oblique direction upward just verifies its positive correlation between the two. That is, as reward *β* increases, this small peak will appear at higher defection temptations. This also predicts that during the game, the reward compensates for the losses suffered by the cooperator when he suffers defection, enabling him to survive. The image in the third row shows a heat map of the frequency of cooperation under the combined influence of alpha and beta. The effects of the two variables influence each other, and it is worth noting the appearance of the triangle in the lower left corner where the cooperation rate varies significantly. That is, when *α* = 0 − 0.1, *β* varies between 0 and 0.2, and the effect on the frequency of cooperation is substantial. This singularity will be discussed in more detail again in subsequent content.

**Fig 2:**
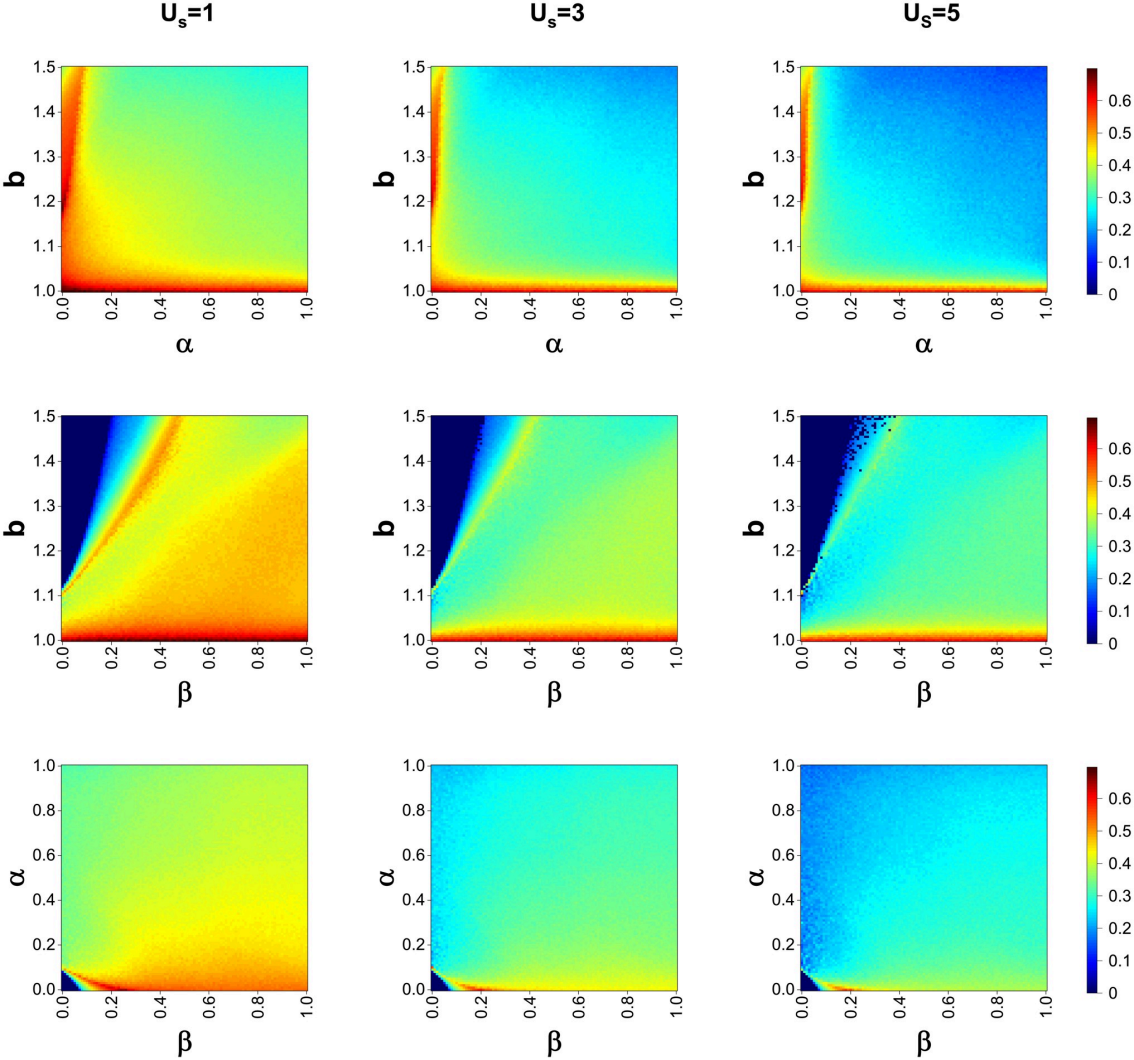
Heat map of the effect of each variable on the frequency of cooperation. The parameters fixed by each line:1.*β* = 0.5, 2.*α* = 0.1, 3.b = 1.1.

**Fig 3:**
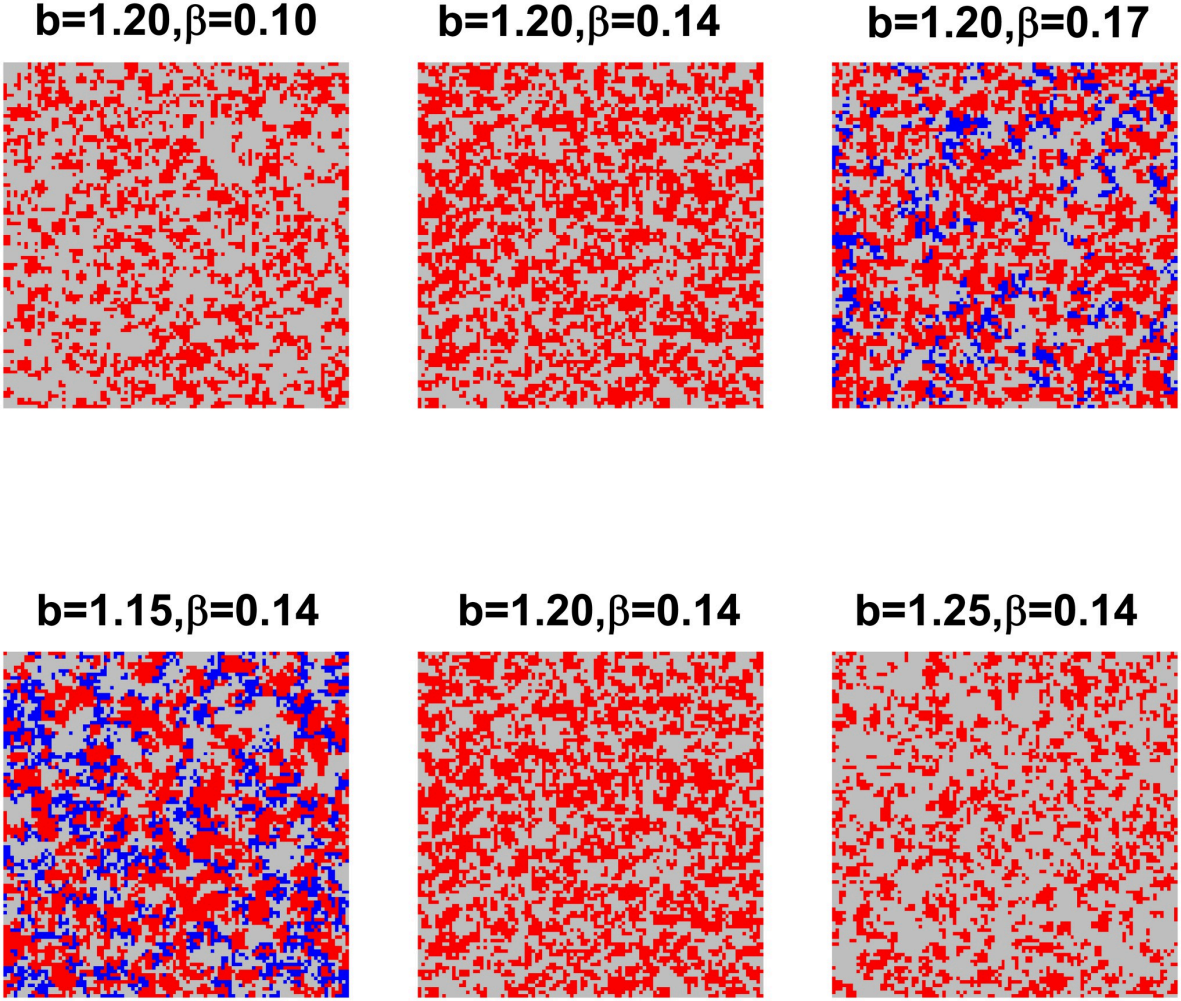
Snapshot of the distribution of player strategies with respect to b and *β*. Where red is cooperation, gray rewards defection, and blue is defection. Other parameter settings: *α* = 0.1, Us = 1.

In order to explore in depth the reasons for the emergence of the high cooperation rate band region in the second row of Fig 2, the distribution of strategies under the relevant parameters is plotted in Fig 3. The strategy distributions in the figure are all obtained after the evolution of the system has stabilized. First in the first row of pictures, we fix the value of b to 1.20. The values of *β* are in turn 0.10, 0.14, and 0.17. A side-by-side comparison reveals that the value of *β* increases significantly from 0.1 to 0.14, the number of cooperators in the group increases significantly, and in this way the group of cooperators starts to become dense. However, as the value of *β* changes from 0.14 to 0.17, due to the increasing proportion of the cooperator group, the group will produce defectors chasing absolute interests again, which eventually leads to the evolution of the group to a state where the three strategies coexist. In the second row of Fig 3, we fix *β* = 1.14. The opposite effect to that of the first row is similarly achieved by changing the size of the defection temptation b. When b = 1.15, the system evolves to the point where the three strategies coexist, however as the temptation to defect grows to 1.20 it instead makes pure defectors disappear. And as b increases to 1.25, defectors with rewarding behaviors in the group increase and clearly outnumber cooperators significantly. Taken together, this analysis suggests that the specific matching combination of defection temptation *b* and reward *β* allowed the system to eventually evolve to a state of higher cooperation rates. And this state happens to be the critical state where defectors have not yet arisen again in the population. The emergence of this critical state is jointly controlled by the two variables mentioned above. That is, a specific combination of defection temptation and reward is required to achieve a higher frequency of cooperators in the population, with all other covariates fixed.

**Fig 4:**
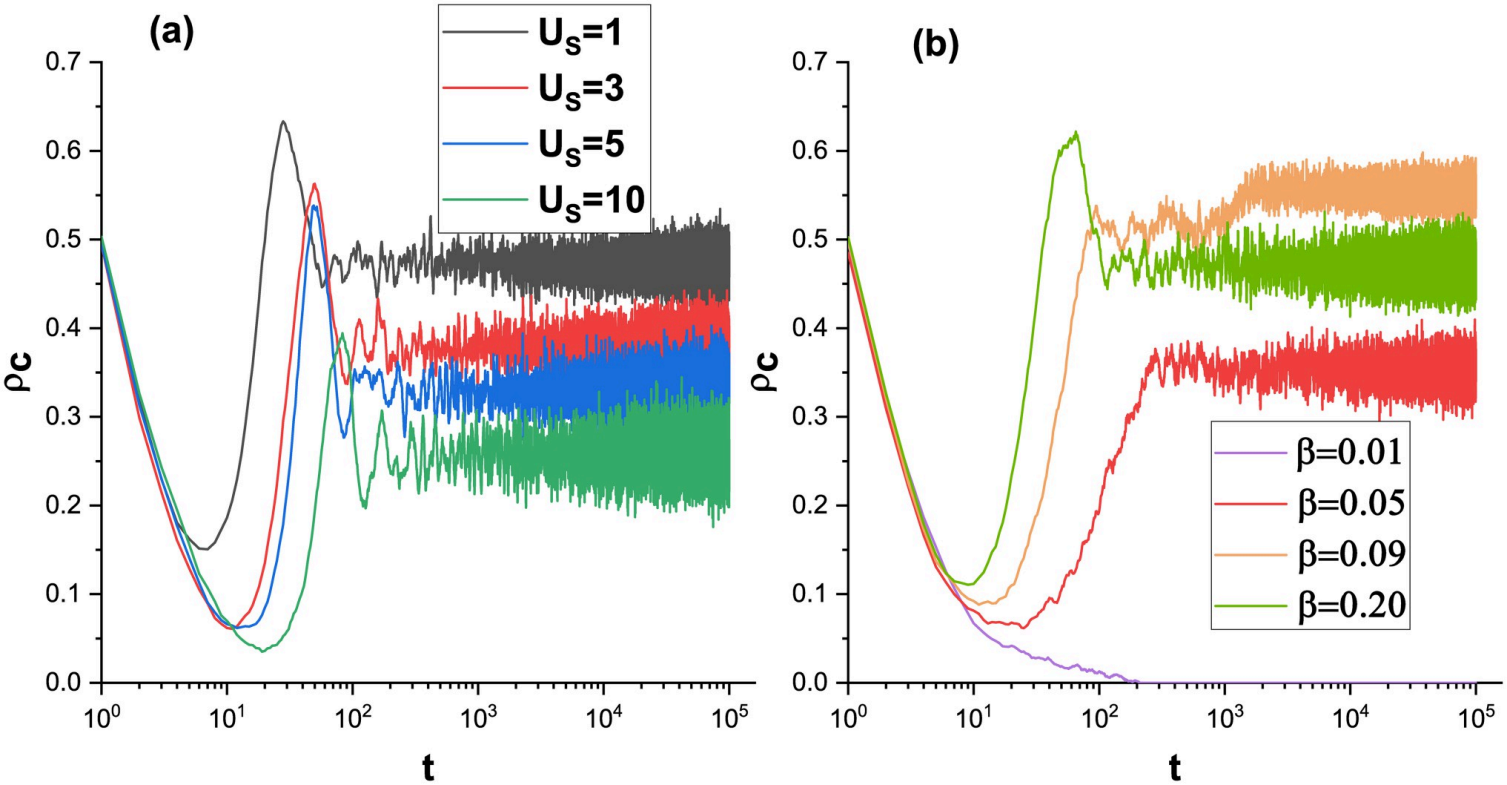
Cooperator frequency *ρ*_*c*_ as a function of time step t. Other parameters: a: *α* = 0.1, *β* = 0.5, *b* = 1.1; b: *α* = 0.05, *Us* = 1, *b* = 1.1.

To study the gradual evolution over time in the system and how it evolves to a steady state, Fig 4 is plotted. In Fig 4a, the variation curves of the cooperator frequency of the system with time step for different survival stress thresholds. The curve in the figure is roughly divided into four parts, the initial declining part is similar to the other mechanisms, and the collaborators are gradually decreasing with the evolutionary process when the mechanism’s role is not yet apparent. Then the bottom point is reached, and the effect of the role of the model in the system begins to show. Comparison of the different curves reveals that a smaller survival stress threshold U causes this nadir to come a little faster and higher. This suggests that more easily reached thresholds make the model more sensitive and effective. The curve then starts to rise gradually, with the number of cooperators coming to the highest point as the system evolves. Since the group of collaborators is not so dense, it is inevitable that there will be some profit-oriented defectors, who make high profits among the collaborators. As a result, the number of collaborators decreases again and eventually reaches a steady state. Meanwhile linking in snapshot Fig 3, the reason why the collaborators in the steady state are maintained actually lies ultimately in rewarding the existence of the special role of the defectors. It seems to become the boundary of communication between the group of defectors and the group of cooperators. Fig 4b plots the collaborator frequency over time for different betas. The purple curve (*β* = 0.01) is overwhelmed by the fact that the amount of reward is too small and the collaborators are not enough to sustain themselves. At the same time, we find that when the reward is small (*β* = 0.05, 0.09), the curve does not show the highest point of the green curve (*β* = 0.20) decreasing and then reaching a steady state. They seem to enter the final state in a much smoother way. Our analysis yields that smaller rewards produce a more stable behavior of hugging cooperators, and that defectors are more unlikely to emerge among them in the subsequent evolutionary process. Of particular note, the orange curve (*β* = 0.0.9) has a higher level of final cooperators than the green curve (*β* = 0.20). This is exceptional, and it will provide a methodology for how how to save costs and increase the frequency of cooperators.

**Fig 5:**
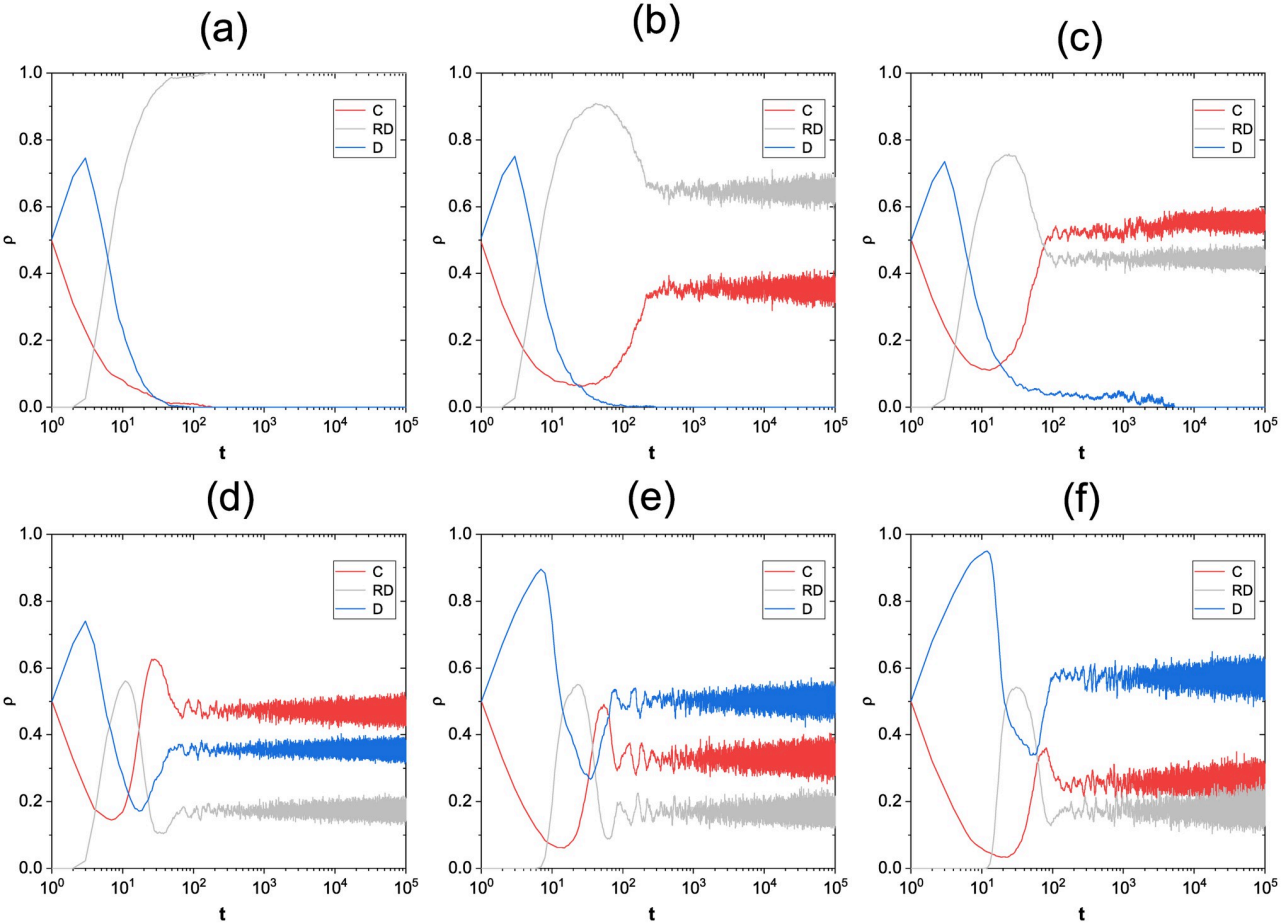
Strategy frequency as a function of time step. The first line fixes the parameters: *α* = 0.05, *Us* = 1, *b* = 1.1; a: *β* = 0.01, b: *β* = 0.05, c: *β* = 0.09 and the second line fixes the parameters: *α* = 0.1, *β* = 0.5, *b* = 1.1; d: *U*_*s*_ = 1, e: *U*_*s*_ = 5, f: *U*_*s*_ = 10.

To further compare the evolution of the three strategies over time, Fig 5 is plotted and yields a clearer picture of how the strategies transform into each other. By looking at subplots a-c of Fig 5,it is clear that the reward *β* received by the cooperator has an impact on the three strategies during evolution. Because the initial state is not set to reward defectors (this is to better fit the real situation, in the initial game defectors because there is no survival pressure so they will not directly lose their own interests to reward others), so the defection strategy from the beginning of the dominant and increase, as it grows to a certain level, the defectors began to face the pressure of survival and have to take the rewarding behavior and change to RD strategy. The number of defectors with rewarding behaviors gradually increases and eventually enters into a dynamic equilibrium with the cooperators, which is affected by the size of *β*. Subfigures d-f were obtained by varying the size of the *U*_*s*_. The environmental stress threshold most directly affects the time step required for a player to start adopting the RD strategy. The RD strategy in subfigure d is initiated significantly earlier than subfigure e and f. This is because with a low survival stress tolerance, players will begin to change their strategies earlier in order to seek a way to survive. And the RD strategies all act as intermediate reconciliation in the evolution process, first increasing and then decreasing, which finally makes the three strategies enter a dynamic equilibrium state.

**Fig 6:**
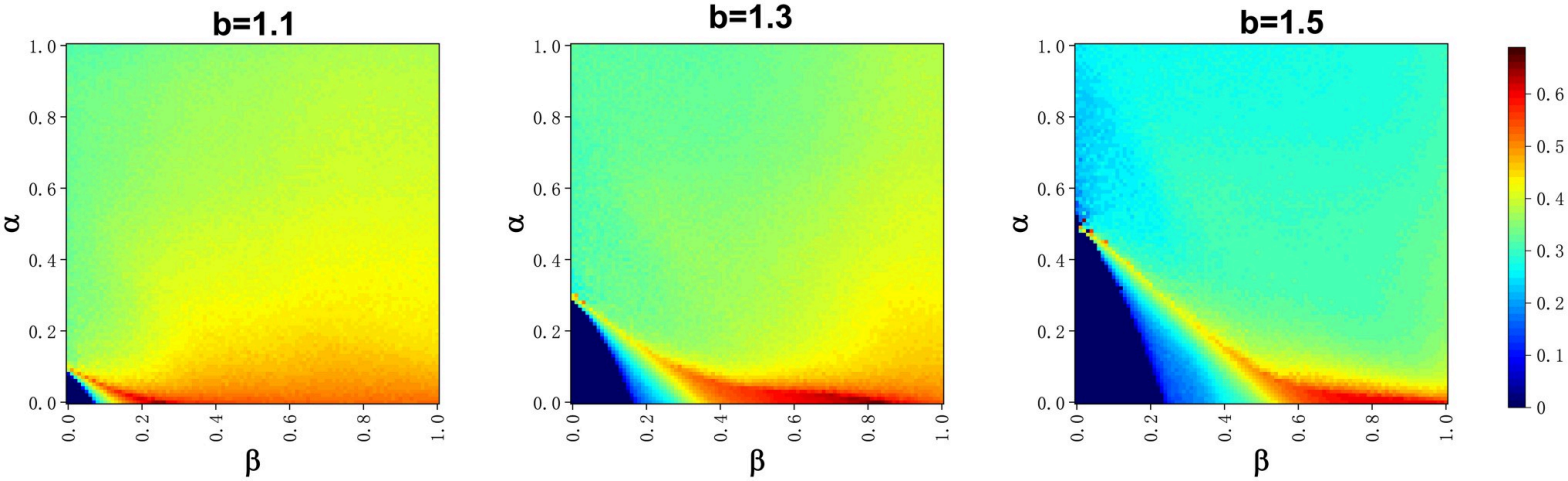
Heatmap of cooperator frequencies for *α* and *β* panels. The parameters of the three horizontal subplots are b = 1.1, 1.3, 1.5.

In the previous discussion of the third row of Fig 2, we found that the color change is more pronounced in the lower left corner of the heat map. In order to investigate this point in depth, we plotted three heatmaps about b changes in Fig 6 by experimental simulation. By comparing the three subgraphs horizontally, we find that the temptation to defect influences the effect of alpha and beta’s on the frequency of cooperators. This is also the same as what we perceive to be the case, where the amount of reward and the cost to be paid when defectors implement rewarding behaviors need to be adjusted according to the size of the defection temptation. Below, we analyze the relationship between alpha and beta specifically, using the rightmost subplot with b = 1.5 as an example. It can be observed in the heatmap that when *α* exceeds 0.5, the effect of *β* on cooperator frequency becomes small, and the change in cooperator frequency during the change of *β* from 0-1 is within 0.2. In the section with *α* less than 0.5, we still find a collaborator frequency heatmap highlighted. That is, when the defection temptation is fixed, the optimal combination of reward beta and cost alpha can still be found. With this ration, defectors in the group receive survival pressure and are able to bear a cost they can accept to reward collaborators, and collaborators are able to survive and sustain themselves because they are funded by this one reward.

**Fig 7:**
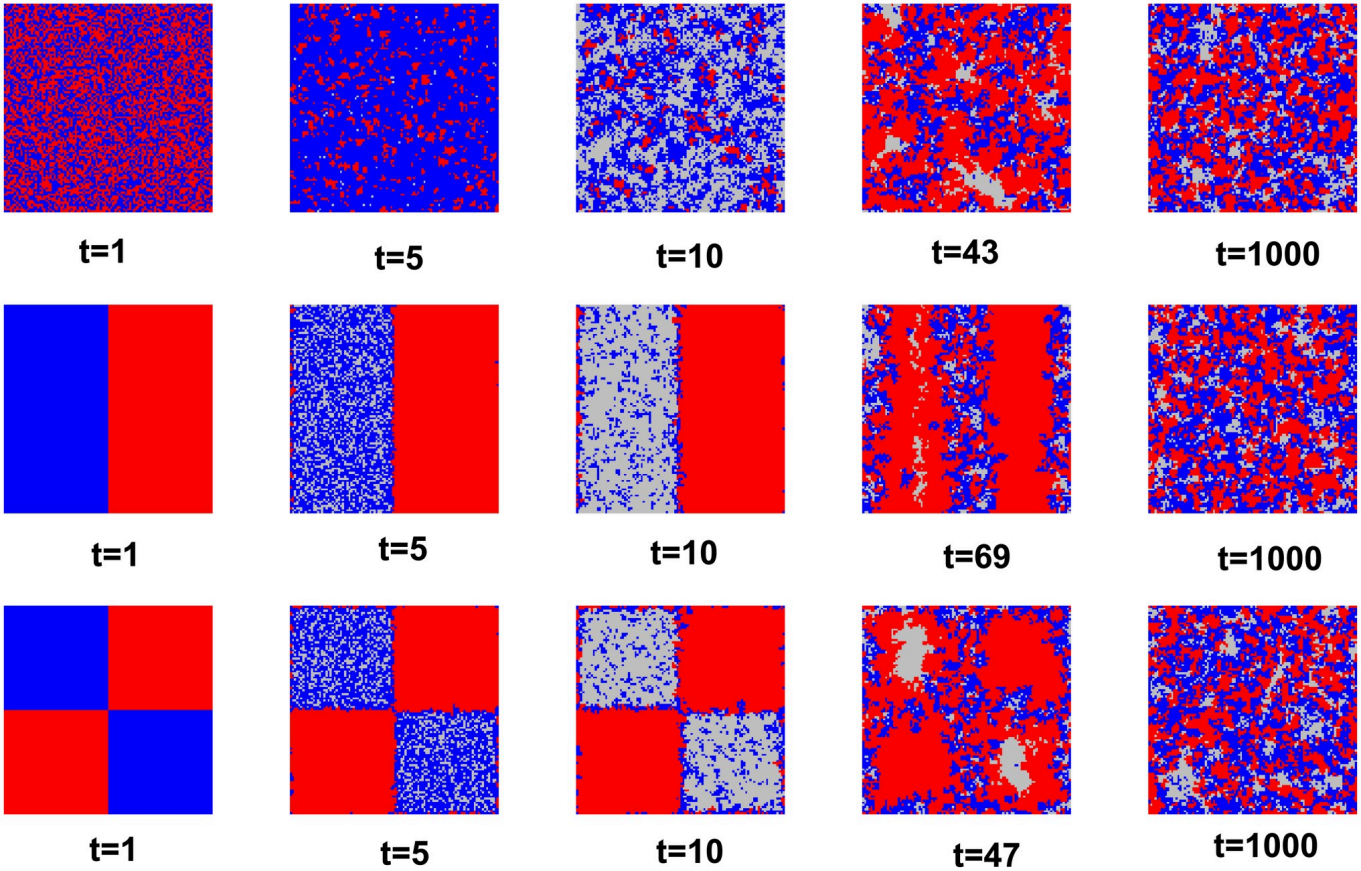
Snapshots of three different initial states. Where the initial states are randomly distributed for cooperators defectors, bipartite, and quadripartite, respectively. Red are cooperators, blue are defectors, and gray are defectors who perform the rewarded behavior. Common parameters: Us = 3, *α* = 0.1, *β* = 0.5, b = 1.1. L = 100.

Taken together with the analysis earlier in the article, the environment (set of neighbors) in which a cooperator in a group is located affects whether or not that cooperator can persist. In order to analyze the effects of spatial structure and neighbor set on system evolution, Fig 7 plots snapshots of system evolution for three different initial states. The fourth column of the figure is selected for the special time-step snapshot, and the other first column, t = 1, shows the initial state of the system evolution. By observing the time-step snapshots, we find that in the process of system evolution, since the initial state does not directly set up defectors with rewarding behaviors, their emergence is more reflective of a kind of behavior produced by defectors under the pressure of survival in the process of system evolution.

In a 100x100 lattice network, 1000 steps would essentially allow the system to evolve to a stable state. We note that when the initial state is randomly distributed, the evolution of the whole system will be accelerated. In the first line of subgraphs, the initial state cooperators defectors are randomly assigned to the network (t = 1), and after the game evolution, the group is dominated by defectors (t = 5). However, this scenario inevitably leads to a gradual transformation of players living deep inside the group of defectors into rewarding defectors by receiving extreme survival pressure (t = 10). Their change in strategy will bring hope that they will be able to attract a significant increase in the number of collaborators (t = 43). And when the number of cooperators increases, attracted by the higher temptation to defect, pure defectors will again appear in the group of cooperators and gain high returns, which will lead to a decrease in rewarding behavior. Eventually the system moves to dynamic equilibrium. Rewarding defectors become an opportunity to attract the emergence of cooperators, but at the same time, they also lay the groundwork for subsequent defector backlash. This evolutionary process will be more pronounced when the initial state separates the group of cooperators from the group of defectors. At t = 10, only the border part of the defectors can rely on high returns without the need for rewarding behavior. In short, this defection strategy with rewarding behavior is more like an excessive strategy of the defectors in the system, through which they squeeze other defectors based on the survival pressure, and when and around the large number of cooperators, they can get rich benefits by canceling the rewarding behavior. However, this kind of fighting behavior between defectors is also the reason for the dynamic emergence and maintenance of cooperators, and in another way, this behavior still promotes the emergence of cooperative behavior.

## Conclusions

Inspired by the competitive behavior of people in the same trade, and different from previous reward models, we propose a model based on the survival pressure of defectors forced to adopt reward behaviors, which will be more in line with the real-life reality where no one voluntarily pays the cost of rewards, and examine its impact on cooperation in the spatial Prisoner’s Dilemma.

The results of the study suggest that defectors in a group are compelled by survival pressure to adopt the rewarded behavior in an attempt to bring about a solution to their dilemma. This behavior will indirectly promote the emergence of cooperators. The smaller the survival pressure threshold *U*_*s*_, the more it reflects that players are more sensitive to the perception of pressure and thus more likely to adopt rewarding behaviors. Then the more effective the mechanism will be in acting on the level of cooperation in the group. In addition, the effect of the payment cost *α* and reward amount *β* of reward behavior on the evolution of the system will be limited by the defector temptation b. And the effect of each parameter on the promotion of cooperation in the system is not linear. That is, when *α* is fixed, b and *β* can still make the level of cooperation optimal in a particular combination. And the same holds true for *α* and *β* when b is fixed.

Rewarding behavior can indeed greatly facilitate cooperation in groups on the ground. However, the cost of adopting the behavior has been limiting it. This paper provides a new perspective to explain the emergence of reward behavior. However, the model is currently only relevant for simulation experiments in lattice networks and is relatively small in size relative to social networks. And it has not been compared with other mechanisms to study the effectiveness of its effects. In the future, it could be extended to other complex networks to explore differences in spatial reciprocity. And the effects of other mechanisms can be incorporated into the research process, combined with a comparative study of the effectiveness of the model. the future, it can be extended to other complex networks to explore the differences in spatial reciprocity.
